# The Impact of Maternal Pre-Pregnancy Body Weight and Gestational Diabetes on Markers of Folate Metabolism in the Placenta

**DOI:** 10.3390/nu10111750

**Published:** 2018-11-13

**Authors:** Jole Martino, Maria Teresa Segura, Luz García-Valdés, M C. Padilla, Ricardo Rueda, Harry J. McArdle, Helen Budge, Michael E. Symonds, Cristina Campoy

**Affiliations:** 1 Early Life Research Unit, Division of Child Health and Obstetrics and Gynaecology, Nottingham NG7 2UH, UK; jolemartino78@gmail.com (J.M.); helen.budge@nottingham.ac.uk (H.B.); 2 EURISTIKOS Excellence Centre for Paediatric Research, University of Granada, 18071 Granada, Spain; msegura@ugr.es (M.T.S.); luzgarciavaldes@hotmail.com (L.G.-V.); ccampoy@ugr.es (C.C.); 3 Department of Obstetrics and Gynaecology, University of Granada, 18071 Granada, Spain; carmenpadvin@gmail.com; 4 Abbott Nutrition, 18004 Granada, Spain; ricardo.rueda@abbott.com; 5 The Rowett Institute of Nutrition and Health, University of Aberdeen, Aberdeen AB24 3FX UK; h.mcardle@abdn.ac.uk; 6 Nottingham Digestive Disease Centre, Biomedical Research Unit, School of Medicine, University of Nottingham, Nottingham NG7 2UH, UK

**Keywords:** body mass index, gestational diabetes, placenta, folic acid

## Abstract

Dietary methyl donors, including folate, may modify the placenta and size at birth but the influence of maternal body weight has not been widely investigated. We therefore examined whether maternal or fetal folate status, together with indices of placental folate transport, were modulated by either maternal pre-pregnancy body mass index (BMI i.e., overweight: 25 ≤ BMI < 30 or obesity: BMI ≥ 30 kg/m^2^) and/or gestational diabetes mellitus (GD). We utilised a sub-sample of 135 pregnant women participating in the Spanish PREOBE survey for our analysis (i.e., 59 healthy normal weight, 29 overweight, 22 obese and 25 GD). They were blood sampled at 34 weeks gestation, and, at delivery, when a placental sample was taken together with maternal and cord blood. Placental gene expression of folate transporters and DNA methyltransferases (DNMT) were all measured. Folate plasma concentrations were determined with an electro-chemiluminescence immunoassay. Food diaries indicated that folate intake was unaffected by BMI or GD and, although all women maintained normal folate concentrations (i.e., 5–16 ng/mL), higher BMIs were associated with reduced maternal folate concentrations at delivery. Umbilical cord folate was not different, reflecting an increased concentration gradient between the mother and her fetus. Placental mRNA abundance for the folate receptor alpha (*FOLR1*) was reduced with obesity, whilst *DNMT1* was increased with raised BMI, responses that were unaffected by GD. Multi-regression analysis to determine the best predictors for placental *FOLR1* indicated that pre-gestational BMI had the greatest influence. In conclusion, the placenta’s capacity to maintain fetal folate supply was not compromised by either obesity or GD.

## 1. Introduction

Folate is an essential cofactor in metabolic pathways that influence DNA methylation patterns, DNA synthesis and cell proliferation [1,2]. It is crucial to the 1-carbon cycle where it acts as a transporter of CH3 and the single carbon donor in one carbon metabolism [3]. Limited dietary availability can contribute to abnormal DNA methylation patterns in mice [4] and, thus, potentially to developmental programming [5]. Fortification of the diet with folic acid has been adopted by many countries to ensure that dietary intake is rarely limited, particularly during pregnancy, in order to prevent neural tube defects when folate requirements can increase because of increased rates of cell division and growth, especially during embryo development [6,7]. Indeed, it is now recommended that, during pregnancy, there should be no upper limit on dietary intake [8]. Pregnant women with high body mass index (BMI) and gestational diabetes (GD) can have inadequate dietary intakes of folate [9,10,11] and lower folate concentrations [12,13]. This could contribute to some of the adverse neonatal outcomes associated with maternal obesity [14], including greater risks of preterm deliveries, neural tube defects and low birth weight [15,16,17].

Cellular uptake of folate is mediated by specific transport mechanisms in the placenta, which include the folate receptor alpha (FOLR1), proton coupled folate transporter (PCFT), and folate carrier (RFC) [18,19]. Of these mechanisms, FOLR1 appears to be the most important, at least as far as preterm births are concerned [20]. Folate is bound to the FOLR1 on the maternal side of the placenta and then transported to the fetal circulation by endocytosis/exocytosis [21]. The impact of maternal obesity on the potential capacity of the placenta to modulate folate status acting through FOLR1 has not been examined extensively in humans. One study in women in Texas found that when gestational weight gain was similar in obese and non-obese women, fetal serum folate concentrations and placental folate transport activity were unaffected [22].

The present study, was designed to explore the effects of a raised maternal pre-pregnancy BMI and/or gestational diabetes on markers of folate placental transport and metabolism, taking into account folic acid status and intake under these different maternal metabolic environments. We hypothesised that as folate has a crucial role in providing methyl donors [3], it can influence gene expression for DNA methyltransferases (DNMT1) and DNMT3A [4]. We therefore examined whether potential changes in placental folate transport resulting from high maternal BMI and/or GD, would modulate gene expression of *DNMT*. Our study was conducted in pregnant women from Spain recruited as part of the PREOBE study in which we have previously shown that placental expression of genes involved in energy sensing and oxidative stress are sensitive to maternal obesity (i.e., BMI ≥ 30 kg/m^2^) and GD [23].

## 2. Materials and Methods

### 2.1. Participants

The participants were part of a longitudinal study on the influence of body composition by maternal genetics and nutrition (PREOBE Excellence Project: P06-CTS-02341) undertaken between 2007 and 2010 and registered with www.ClinicalTrials.gov, (NCT01634464) [24,25,26] for which full details have already been published (and are summarised in Table 1 [23]). It was conducted according to the guidelines in the Declaration of Helsinki and all experimental procedures were approved by the Ethics Committees for Granada University, San Cecilio University Hospital and the University of Nottingham. Witnessed, written informed consent was obtained from all participants before their study inclusion and they were assured of anonymity. Anthropometric assessments of mothers and newborns were undertaken following the standards established by the Spanish Society of Gynaecology and Obstetrics, the Fetal Foundation and the Spanish Association of Paediatrics. Gestational Diabetes was diagnosed according to the Spanish consensus protocol established by the *Grupo Español de Diabetes y Embarazo (GEDE) of the* Gynecology and Obstetricians Society, followed by Andalusian pregnant women general practitioners [27]. The O’Sullivan test was performed on all pregnant women between 24–28 weeks of pregnancy, as screening for gestational diabetes. If glucose was ≥140 mg/dL an oral glucose tolerance test (OGTT) with 100 g of glucose loading was performed. Gestational diabetes was diagnosed if two or more glucose values met or exceeded the following values fasting <105 mg/dL, one hour <190 mg/dL, two hours <165 mg/dL, three hours <145 mg/dL. Women with plasma glucose ≥200 mg/dL after O’Sullivan test were diagnosed of gestational diabetes and OGTT was not performed. The O’Sullivan screening test was also performed in the first trimester in high risk pregnant women (maternal age >35 years; BMI > 30 kg/m^2^, previous gestational diabetes or other glucose metabolic alterations, previous obstetric results with an indication of undiagnosed gestational diabetes (e.g., *foetal macrosomy*), family history of diabetes mellitus, ethnic risk groups (Afroamericans, Asiatic-Americans, Hispanic, Indio-Americans))

As shown in Table 1, the subpopulation of 135 participants whose placentas underwent molecular analysis in Nottingham, for which a majority that gave birth at full term i.e., c. 39 weeks [23], with the exception of GDO for which 50% underwent caesarean section delivery. The number of participants per group were, therefore, 59 normal weight women (18 ≤ pre-pregnancy BMI < 25 kg/m^2^ (N)), 29 overweight women (25 ≤ BMI < 30 kg/m^2^ (OW)) and 22 obese women (BMI ≥ 30 kg/m^2^ (O)). Furthermore, the 25 mothers with GD were subsequently classified according to their BMI as normal weight GD (pre-pregnancy BMI < 25 kg/m^2^ (GDN), *n* = 14) and as obese GD (pre-pregnancy BMI ≥ 30 kg/m^2^ (GDO), *n* = 11). There were no effects of the sex of the baby, route of delivery or gestational age on any of the measurements reported in this study.

### 2.2. Maternal Nutrient Intake

Information about maternal nutrient intake was collected during late gestation (34–40 weeks) using standardised 7 day dietary records given to the participants during the second visit (34th gestational week). Each participant was given verbal and written instructions by the investigator on how to record food and drinks consumed during the 7 day recording period and a booklet of common food items and mixed dishes to facilitate estimation of portion sizes. Around the time of delivery, food records were reviewed with each mother by a nutritionist for completeness and accuracy of food description and portion sizes. Nutritional data were analysed for nutrient intake by using a nutritional software program (CESNID 1.0: Barcelona University, Spain) based on validated Spanish food tables (“*Tablas de composición de alimentos del CESNID*”) [29] and took account of those products fortified with folic acid.

### 2.3. Collection and Analysis of Blood Samples

Maternal venous blood was collected at 34 weeks of gestation and during labour (N: *n* = 59; OW: *n* = 29; O: *n* = 22; GDN: *n* = 14; GDO: *n* = 11). Umbilical venous blood samples (N: *n* = 33; OW: *n* = 15; O: *n* = 12; GDN: *n* = 7; GDO: *n* = 7) were collected within 30 min after placental delivery from a double-clamped section of umbilical cord. EDTA and serum collection tubes were used (Vacutainer^®^ Refs: 368857 and 367953) for haematological assessment and biochemical analyses respectively. There were no differences in any of the measurements performed on the placenta between those women from whom blood was sampled and those in whom this could not be achieved e.g., born at a time when sampling could not be undertaken.

Blood samples for serum preparation were held at 4 °C for 15 min to allow blood clotting, centrifuged at 3500 rpm for 10 min at 4 °C, and the serum fraction transferred into sterile tubes. Samples were stored at 4 °C for same-day analyses or at −80 °C for further analysis. Serum folate was determined by an electro-chemiluminescence immunoassay with the automatic analyser Elecsys 2010, and the analytical kit No. E170 (Roche, Neuilly sur Seine, France).

### 2.4. Collection of Placenta Samples

Placenta samples from all participants were collected and weighed immediately after delivery as previously published [23]. Visual inspection of the placenta for necrosis or any other abnormality was undertaken by experienced clinicians. A representative 0.5 × 0.5 × 0.5 cm (200 mg) sample was excised from the middle of the radius (distance between the insertion of the umbilical cord and the periphery) of each placenta, rinsed twice with saline solution (0.9% NaCl) and immediately placed into sterile 1.5 mL microtubes (Greiner Bio One, Monroe, NC, USA) containing RNAlater solution (Qiagen Ltd., Crawley, UK). All samples were frozen under RNase free conditions using liquid nitrogen before storage at −80 °C for later analysis in Nottingham.

### 2.5. Laboratory Analysis

Total RNA was extracted from 100 mg of maternal placenta tissue using 200 µL of chloroform per 1 mL of TRI reagent solution (Sigma Chemical Co., Poole, UK) and RNeasy extraction kit (Qiagen Ltd., Crawley, UK) as previously published [23]. RNA quality was assessed by gel electropheresis. Two µg RNA was used to generate 20 µL cDNA using High Capacity RNA-to-cDNA kit (Applied Biosystems, Foster City, CA, USA). Negative control RT samples lacking Enzyme Mix (-RT) were included for each sample. Real-time PCR using 15 µL of reactions consisting of 4.5 µL diluted 1:10 cDNA, 3.0 µL (final concentration of 250 nM) gene specific primers (Table 2, Sigma-Aldrich, St. Louis, MO, USA), and 7.5 µL of SYBR Green mastermix (Thermo Scientific, ABgene Ltd., Epson, UK) were performed. Duplicate samples were run for 40 cycles with negative controls in 96-well plates using the Techne Quantica Thermocycler (Techne Inc., Barloword Scientific, Stone, UK). Ten-fold serial dilutions of cDNA for each gene were used to generate standard curve analysis and only experiments with *R*^2^ > 0.985 were included. CT measurements, calculated by 2^−ΔCt^ method [30], were used for mRNA expression. A range of housekeeping genes were used including *ACT8*, *18S* and *B2M*, for which 18S ribosomal RNA was used as the optimal housekeeping gene for data normalisation, as previously published [23]. It was the most stable housekeeping examined (i.e., N: 0.37 ± 0.08; OW 0.70 ± 0.28; OB 0.56 ± 0.11; GDL: 0.75 ± 0.14; GDO: 0.13 ± 0.02 a.u.) and the use of other reference genes made no difference to the results.

### 2.6. Statistical Analysis

All statistical evaluations were performed by using IBM SPSS v20.0 statistical software for Windows (IBM Corp., Armonk, NY, USA). To assess the data for normality, a Kolmogorov–Smirnov test was performed, where *p* values >0.05 indicated that the data were normally distributed. Thereafter, appropriate parametric, or non-parametric, tests were used to analyse the effects of maternal overweight and obesity as follows: (1) comparisons of blood folate concentration at each sampling age between comparable groups (i.e., N vs. OW, or O, or GDN; GDN vs. GDO) were made by independent *t*-test; whilst (2) differences in gene expression were determined by using Mann-Whitney test. Categorical data were analysed using Chi-square test of independence. Continuous data (i.e., gene expression and folate concentrations) presented are expressed as means with their standard errors (SEM), with *p* values < 0.05 deemed to represent statistical significance. For all analyses undertaken, there was no effect of foetal sex or route of delivery.

Association between continuous variables were also assessed using multiple linear regression analysis. It included the following three models: The first was adjusted for the a priori confounder of pre-gestational BMI; the second, for both BMI and maternal glucose at 34 weeks of gestation and the third for BMI, maternal glucose and folate at 34 weeks gestation.

## 3. Results

### Maternal Folate Status and Adaptations within the Placenta

**Figure 1:**
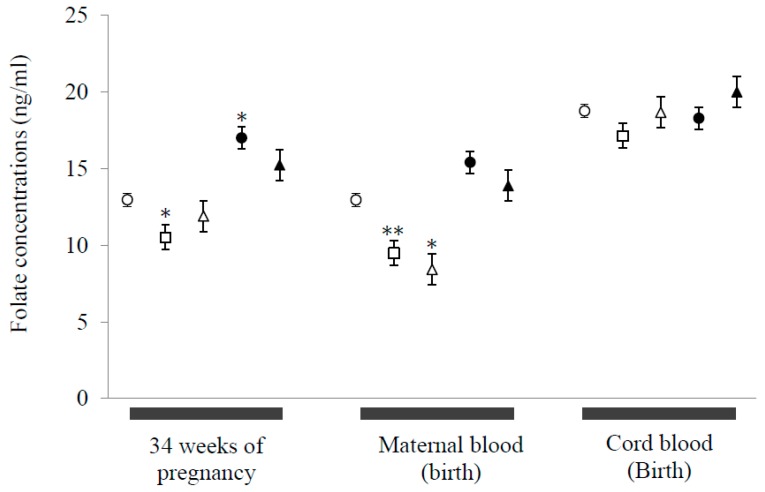
Effects of maternal pre-pregnancy BMI and gestational diabetes on maternal and neonatal folate serum concentrations. Maternal samples were taken at 34 weeks gestation and at term/delivery i.e., c. 39 weeks of pregnancy, neonatal samples were taken from cord blood at birth. Open circles: normal weight (N: maternal, *n* = 59; cord, *n* = 33); open squares: overweight (OW: maternal, *n* = 29; cord, *n* = 15); open triangles: obese (O: maternal, *n* = 22; cord, *n* = 12); closed circles: gestational diabetic, normal weight (GDN: maternal, *n* = 14; cord, *n* = 7); closed triangles: gestational diabetic, obese women (GDO: maternal, *n* = 11; cord, *n* = 7). Values represent means ± S.E.M. Statistical differences between groups denoted at each time point by *, ** correspond to *p* < 0.05, *p* < 0.01 respectively compared to normal weight control group (independent t test for continuous variables).

Amongst those participants for whom placental analysis was undertaken and who reported daily intakes of folate (400 µg dietary folate equivalents/day) and iodine supplements at 24th week of pregnancy, there were no differences in folate or vitamin B12 consumption between groups (Table 3). Folate concentrations in all participants in the present study were within the normal range (6–20 ng/mL) with no evidence of folate deficiency (defined as <5 ng/mL: Figure 1) [7]. GDN women exhibited the highest serum folate concentrations at 34 gestational weeks compared to the normal weight group (Figure 1). This could reflect dietary and related advice given to this sub-group, in which mean folate and vitamin B12 intakes were also highest (Table 3). There was no significant effect of taking folate supplements in late gestation on maternal folate concentrations at 40 weeks of gestation. A marked decrease in folate concentrations in late pregnancy was observed in both OW and O women (Figure 1). Cord blood folate concentrations were not different between groups, reflecting an increased difference between maternal folate at 40 gestational weeks and umbilical cord folate with raised maternal BMI (i.e., Δ folate–N: 5.80 ± 0.81; OW: 7.66 ± 0.82; O: 9.01 ± 0.90 ng/mL (*p* < 0.05)). There was also a positive correlation between maternal and newborn plasma folate in the N (*r*^2^ = 0.38; *p* < 0.0001) and GDN (*r*^2^ = 0.81; *p* = 0.015) groups at 34 weeks gestation and in the N (*r*^2^ = 0.19; *p* = 0.013) and OW (*r*^2^ = 0.48; *p* = 0.006) groups at 40 weeks gestation. No infants exhibited any spinal or neural abnormalities.

Placentas from obese women showed a significantly lower expression of *FOLR*1, a response that was unaffected by GD (Table 4). Multi-regression analysis of predictors for placental *FOLR1* gene expression, indicated that pre-pregnancy BMI had the greatest influence (Table 5), suggesting an effect present in early gestation. Gene expression of placental methylenetetrahydrofolate reductase (NAD(P)H) (*MTHFR*), the enzyme that catalyses the synthesis of 5-MTHF, did not significantly change between BMI groups, but was raised in obese women with GD (Table 4). Whilst placental *DNMT3A* gene expression was similar between groups, mean mRNA expression of *DNMT1* was higher in overweight and women (Table 4). No significant correlations were found between placental gene expression and plasma folate.

## 4. Discussion

We show that although maternal folate concentrations were reduced with raised maternal pre-pregnancy BMI near to term, this was not apparent with GD, suggesting that the dietary advice provided improved folate status irrespective of any placental adaptations, as seen with obesity. In those women with raised pre-pregnancy BMI, there was no detectable reduction in cord blood folate and all infants were healthy at term. This suggests appropriate adaptation in folate transfer across the placenta, that has been suggested to include a downregulation of *FOLR1* gene expression in the placenta with obesity [19]. The results from our study support such a proposal as gene expression for the other two transporters, *PCFT* and *RFC,* were unaffected. Raised mRNA abundance of *FOLR1,* if translated to protein, and accompanied with a decline in maternal folate concentration would result in the maintenance of cord blood folate as we observed at term with obesity. Alternatively mRNA transcripts of this folate transporter could undergo further functional modifications, that modulate the placentas capacity to promote folate transport [31] and, hence, protect normal foetal folate status as seen in women from Texas, USA [22].

The negative association between raised pre-pregnancy BMI and folate status did not appear to be related to dietary inadequacy in these women unlike those described by others [9,10,11]. It is possible that other adaptations to a high pre-pregnancy BMI, such as hormonal changes in pregnancy and endocrine modifications, could contribute [6]. It is possible that adaptations within the maternal microbiome could impact on maternal folate status [32], as has been shown in the rat during late gestation when manipulating macronutrient intake [33]. Interestingly, obesity with GD resulted in raised placental *MTHFR* gene expression that could ultimately inhibit intracellular homocysteine release by promoting 5-MTHF synthesis [34,35,36]. Enhanced folate catabolism by the placenta would also limit homocysteine accumulation within the trophoblast, thereby avoiding foetal complications in women with obesity and/or GD [35,37].

As folate deficiency has been associated with increased placental S-adenosyl-methionine to S-adenosyl-homocysteine ratio [38,39] and decreased genomic DNA methylation [40,41,42,43], reduced blood folate with obesity could lower the availability of placental SAM, which is used by DNMTs to methylate DNA [2,44,45]. Changes in gene expression for *DNMT1* could be important in this regard as it transcribes the enzyme required for the maintenance of DNA methylation [46] although this was not measured in the present study. DNMT1 is also essential for cellular development [46] and our observation of raised placental gene expression with increased BMI could impact on these processes even though *DNMT3A* was unaffected. These divergent responses could reflect their contrasting roles, with DNMT3A catalysing de novo DNA methylation during early development, whilst DNMT1 is responsible for the maintenance of DNA methylation throughout all development stages [4]. The decreased maternal folate with raised pre-pregnancy BMI and higher mRNA abundance of *DNMT1* in the placenta with similar cord blood folate concentrations, could be indicative of a compensatory response in order to maintain adequate methylation status, for which no differences were found when measured in five subjects per study group [47]. Overall the relatively small sample size was utilised in our study, which might benefit from being undertaken in larger groups of women of different ethnicity. Such a broader study could be combined with a more detailed assessment of those epigenetic adaptations which remain to be clarified [48].

## 5. Conclusions

In conclusion, pregnancies in Spanish women with a high BMI and GD are differentially associated with changes in maternal serum folate in late gestation, which suggests a protective role by the placenta of the foetus, further supporting the need to ensure optimal dietary folate intake [8].

## Figures and Tables

**Table 1:** Summary of maternal age, body and birth weights of all participants.

	N (*n* = 59)	OW (*n* = 29)	O (*n* = 22)	GDN (*n* = 14)	GDO (*n* = 11)
Maternal characteristics					
Age at delivery (years)	30.4 ± 4.5	30.9 ± 7.2	29.0 ± 4.7	33.1 ± 4.1 *	34.7 ± 4.3 **
Height (cm)	162.9 ± 5.7	162.5 ± 6.4	162.7 ± 6.2	159.3 ± 3.9	160.5 ± 6.0
Pre pregnancy BMI (kg/m^2^)	21.8 ± 1.8	27.8 ± 2.2 ***	32.5 ± 2.6 ***	22.4 ± 1.8	35.5 ± 4.9 ***
BMI at 34 weeks (kg/m^2^)	26.6 ± 2.6	31.3 ± 2.4 ***	35.4 ± 2.4 ***	25.9 ± 2.6	36.4 ± 4.1 ***
GWG 0–34 weeks (kg)	12.6 ± 4.3	9.9 ± 4.6 **	7.3 ± 5.1 ***	9.0 ± 5.6 **	2.2 ± 7.8 ***
Preterm (<37 gestational weeks) (*n*)	2 (3%)	1 (3%)	2 (9%).	1 (14%)	4 (27%) *
Male newborn (%)	53	40	62	57	73
Number of caesarean section (%)	12	26	38	25	50
Number on supplements *^+^*	45	26	22	12	9
Infant characteristics					
Newborn weight (g)	3292 ± 410	3230 ± 587	3454 ± 549	3374 ± 402	3415 ± 549
Placental weight (g)	469 ± 120	495 ± 135	531 ± 114 *	498 ± 134	476 ± 93
Placental: birth weight ratio	0.14 ± 0.03	0.16 ± 0.05	0.16 ± 0.04	0.15 ± 0.04	0.14 ± 0.02
Gestational age (weeks)	39.2 ± 1.0	39.4 ± 1.6	39.3 ± 1.7	39.3 ± 1.3	38.8 ± 1.3

Pre: pregestational; BMI: body mass index; GWG: gestational weight gain during the first 34 gestational weeks based on 2009 IOM guidelines for each category [28]. ^+^ reported taking folate and iodine supplements at 24th week of gestation. Values are means ± SD or categorical data as appropriate; n: number of women per group; gw: weeks of gestational. Statistical differences: * *p* < 0.05, ** *p* < 0.01, *** *p* < 0.001 compared to normal w eight group (Chi-square test or t-independent test for continuous variables; chi-square test for categorical variables). Based on their pre- pregnancy weights they were classified as being of normal weight (N), overweight (OW), obese (O), gestational diabetic, normal weight (GDN) or gestational diabetic, obese (GDO) pregnant women (Martino et al., 2016) [23].

**Table 2:** Summary of primers used together with qPCR product and conditions.

Target Gene	Forward Primer Sequence	Reverse Primer Sequence	Product Size (bp)	Temp (°C)
*FOLR1*	CACTCCCTGCCTGTCTCC	TCTGCTCTGCTCTACACTCC	80	59
*PCFT (SLC46A1)*	ATGCAGCTTTCTGCTTTGGT	GGAGCCACATAGAGCTGGAC	100	60
*RFC (SLC19A1)*	CAGCATCTGGCTGTGCTATG	TGATGGTCTTGACGATGGTG	161	59
*MTHFR*	TCCCGTCAGCTTCATGTTCT	TGTCGTGGATGTACTGGATGA	116	59
*DNMT1*	TTCTTCGCAGAGCAAATTGA	CGTCATCTGCCTCCTTCATGG	210	57
*DNMT3A*	AAGCCTCAAGAGCAGTGGAA	AAGCAGACCTTTAGCCACGA	190	59

*FOLR1*: folate receptor alpha; *PCFT*: proton coupled folate transporter; *RFC*: reduced folate carrier; *MTHFR*: methylenetetrahydrofolate reductase; *DNMT1*: DNA methyl transferase-1; *DNMT3A*: DNA methyl transferase-3 alpha. bp, base pairs.

**Table 3:** Mean maternal 7 day dietary intake of folate and vitamin B12 between 34–40 weeks gestation for each group of women whose body weight category was defined according to their pre pregnancy BMI.

Maternal Intake (μg DFE/day)	N (*n* = 37)	OW (*n* = 15)	O (*n* = 8)	GDN (*n* = 11)	GDO (*n* = 6)
Folate	298 ± 12	258 ± 18	260 ± 46	342 ± 33	299 ± 53
Vitamin B12	5.8 ± 0.5	4.7 ± 0.5	5.3 ± 0.9	10.1 ± 4.1	5.3 ± 1.2

DEF, dietary folate equivalents. Normal weight: N; overweight: OW; obese: O, gestational diabetic, normal weight: GDN and gestational diabetic, obese: GDO. Values are means ± SD.

**Table 4:** Effects of maternal body mass index on gene expression markers of folate transport and metabolism and DNA methylation in placenta of normal weight (N), overweight (OW), obese (O), gestational diabetic, normal weight (GDN) and gestational diabetic, obese (GDO) pregnant women.

Pathway	NCBI Sequence	Target Gene	N (*n* = 59)	OW (*n* = 29)	O (*n* = 21)	GDN (*n* = 14)	GDO (*n* = 11)
Folate transport and metabolism	NM_016725.2	*FOLR1*	1.0 ± 0.9	0.8 ± 0.6	0.5 ± 0.3 *	0.6 ± 0.3	0.5 ± 0.3 *
	NM_080669.4	*PCFT* ^ψ^	1.0 ± 0.6	1.0 ± 0.5	1.1 ± 0.6	0.6 ± 0.7	0.8 ± 0.5
	NM_006996.2	*RFC* ^ψ^	1.0 ± 0.8	0.9 ± 0.5	1.0 ± 0.7	0.8 ± 0.5	0.7 ± 0.5
	NM_005957	*MTHFR*	1.0 ± 0.9	1.0 ± 0.9	0.8 ± 0.6	1.0 ± 0.7	1.5 ± 0.7 *
DNA methylation	NM_001130823	*DNMT1*	1.0 ± 1.1	1.8 ± 1.1 **	1.5 ± 1.2	1.8 ± 1.5	0.5 ± 0.5
	NM_022552.4	*DNMT3A*	1.0 ± 0.9	1.1 ± 1.2	0.7 ± 0.5	0.9 ± 0.9	0.6 ± 0.3

Data expressed relative to housekeeping gene (ribosomal 18S RNA), normalised to the control group to give the fold change. *n* = women/group. Data are non-parametric and represent mean ± SD Statistical differences: * *p* < 0.05, ** *p* < 0.01 compared to normal weight group (Mann Whitney test). The abundance of genes denoted by ^ψ^ were measured in a representative selection of 20 N women as insufficient mRNA was not available for all samples. *FOLR1*: folate receptor alpha; *PCFT*: proton coupled folate transporter; *RFC*: reduced folate carrier; *MTHFR*: methylenetetrahydrofolate reductase; *DNMT1*: DNA methyl transferase-1; *DNMT3A*: DNA methyl transferase-3 alpha.

**Table 5:** Association between placental gene expression of folate receptor alpha (*FOLR1*) and different predictors in control, overweight and obese pregnant women with or without gestational diabetes (*n* = 135).

Linear Regression Model	*B* (95% CI)	*SE B*	β	*p*
Model 1 ^ψ^				
Maternal pre-BMI	−0.029 (−0.051, −0.007)	0.011	−0.214	0.009
Model 2 ^ψψ^				
Maternal pre-BMI	−0.032 (−0.054, −0.009)	0.011	−0.230	0.006
Maternal glucose (34 gw)	0.004 (−0.002, 0.01)	0.003	0.107	0.194
Model 3 ^ψψψ^				
Maternal pre-BMI	−0.033 (−0.056, −0.011)	0.012	−0.241	0.004
Maternal glucose (34 gw)	0.005 (−0.001, 0.01)	0.003	0.131	0.116
Maternal folate (34 gw)	−0.019 (−0.043, 0.005)	0.012	0.128	0.124

^ψ^ Adjusted for the *a priori* confounders pre-pregnancy BMI; ^ψψ^ adjusted for the *a priori* confounders pre-pregnancy BMI and maternal glucose at 34 weeks of gestation (gw); ^ψψψ^ Adjusted for the *a priori* confounders pre-pregnancy BMI, maternal glucose at 34 gw and maternal folate at 34 gw. *B*: unstandardised beta; 95% CI: 95% Confidence intervals; *SE B*: Standard error of unstandardised beta; β: standardised beta (β).
